# Painful cutaneous nodules with a history of uterine fibroids

**DOI:** 10.1016/j.jdcr.2026.03.055

**Published:** 2026-04-07

**Authors:** Garrett Kraft, Laszlo Karai, Miesha Merati

**Affiliations:** a Dr. Phillip Frost Department of Dermatology and Cutaneous Surgery, University of Miami, Miami, Florida; b Global Pathology, Miami, Florida; c Miami Dermatology & Mohs Surgery, Miami, Florida

**Keywords:** cutaneous leiomyoma, cutaneous nodules, hereditary cancer syndrome, hereditary leiomyomatosis and renal cell cancer syndrome, Reed syndrome

## Case description

**Fig 1:**
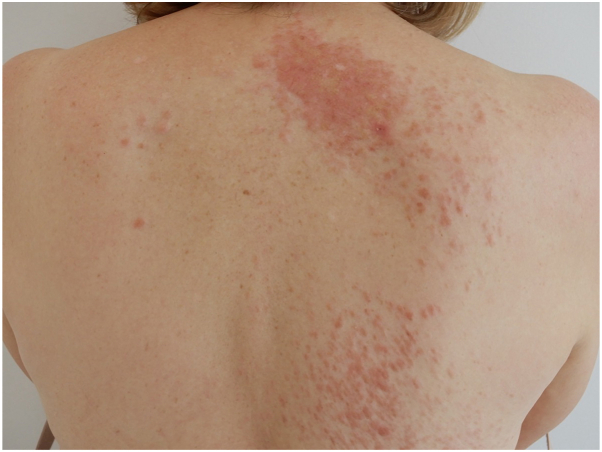
Clinical image of the posterior trunk with multiple coalescing erythematous nodules, forming plaques.

**Fig 2:**
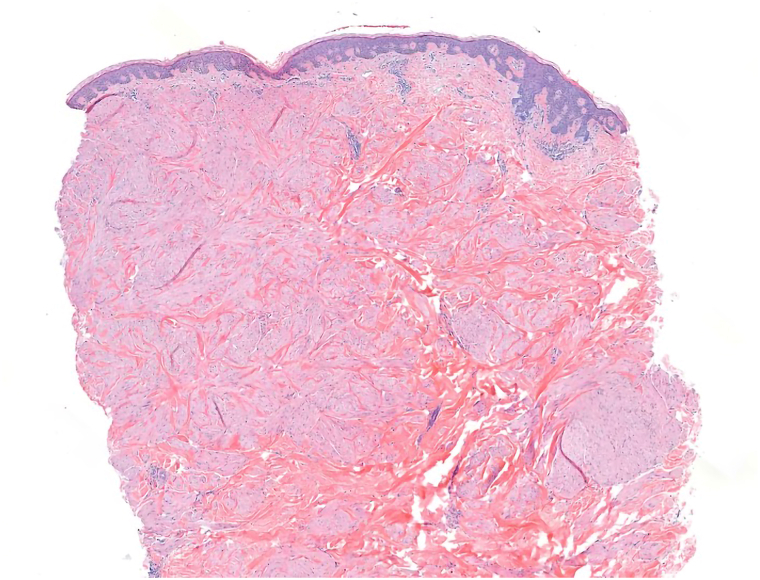
Histopathological image showing a dermal proliferation of interlacing spindle cells with eosinophilic cytoplasm and blunt-ended nuclei (hematoxylin-eosin, original magnification 20×).

A 60-year-old woman with a history of uterine fibroids and cutaneous sarcoidosis presented for evaluation of numerous painful cutaneous nodules on the face and posterior trunk for many years. She described the lesions as painful on direct contact and reported receiving no prior treatments. The patient underwent a hysterectomy for significant fibroid burden and reported an extensive family history of uterine fibroids. Examination was notable for multiple painful red nodules coalescing into plaques (Fig 1). A skin biopsy was performed on her face and back. Histopathology demonstrated a well-circumscribed dermal proliferation of interlacing fascicles of spindle cells with eosinophilic cytoplasm and blunt-ended nuclei (Fig 2).

**Question: What is the most likely diagnosis?**

**A.** Familial multiple lipomatosis
**B.** Hereditary leiomyomatosis and renal cell cancer
**C.** Sarcoidosis
**D.** Neurofibromatosis type I
**E.** Brooke-Spiegler syndrome

### Answer discussion

The correct answer is **B**, hereditary leiomyomatosis and renal cell cancer (HLRCC).

HLRCC, also known as Reed syndrome, is a rare genetic disorder caused by an autosomal dominant mutation in the fumarate hydratase (FH) gene.^1^^,^^2^ Definitive diagnosis is confirmed by a positive FH mutation test.^3^ Individuals with this mutation are susceptible to the development of cutaneous leiomyomas, early-onset uterine leiomyomas, and an aggressive form of type 2 papillary renal cell cancer.^2^ This patient represents a typical case of HLRCC with significant reduction in quality of life secondary to pain. Management of cutaneous leiomyomas depends on symptom burden. Solitary symptomatic lesions may be treated with surgical excision, while more extensive disease can be managed with cryotherapy or laser therapy. Medications that may reduce pain include calcium channel blockers, alpha blockers, nitrates, antidepressants, and antiepileptic agents.^4^ The lifetime risk for renal cell carcinoma in patients with HLRCC is estimated to be 15% to 30%.^2^^,^^3^ Given the high risk for metastasis, early identification and diagnosis are essential for achieving good outcomes and screening family members who may be affected.^3^ Studies support previous consensus recommendations of annual renal imaging screening by magnetic resonance imaging (MRI) (preferred) or computed tomography, starting at age 8-10, to enable early renal cell cancer detection in patients with HLRCC.^5^

The biopsy was consistent with classic histopathologic features of HLRCC. Further workup with germline FH gene sequencing was performed in this patient given the personal and family history of cutaneous and uterine leiomyomas and confirmed the diagnosis of HLRCC. The patient was subsequently given instructions for annual screening by MRI given the elevated lifetime risk of renal cell carcinoma and her first MRI screening was normal. The patient denied any known family history of renal cell carcinoma, although family members had not undergone clinical screening or genetic testing.

Given the autosomal dominant inheritance pattern of HLRCC, first-degree relatives of affected individuals should be referred for genetic counseling and consideration of FH mutation testing. Individuals with HLRCC who have an intact uterus should undergo annual gynecologic evaluation with periodic pelvic imaging (MRI or ultrasound) to monitor leiomyoma burden which may have implications for reproductive planning.^4^ Dermatologists play a critical role in identifying HLRCC, as recognition of cutaneous leiomyomas may be the first opportunity to diagnose the syndrome and initiate renal cancer surveillance.

## Conflicts of interest

None disclosed.
